# Complete genome sequence of a *Salimicrobium* sp. PL1-032A isolated from the pink hypersaline Pearse Lakes, Rottnest Island, Western Australia

**DOI:** 10.1128/mra.00361-24

**Published:** 2024-08-20

**Authors:** Crystal E. Young, Hussain Alattas, Colin Scott, Daniel V. Murphy, Ravi Tiwari, Wayne G. Reeve

**Affiliations:** 1 Bioplastic Innovation Hub, Food Futures Institute, Murdoch University, Murdoch, Western Australia, Australia; 2 School of Medical, Molecular and Forensic Sciences, Murdoch University, Murdoch, Western Australia, Australia; 3 CSIRO Environment, Black Mountain Science and Innovation Park, Canberra, Australia; SUNY College of Environmental Science and Forestry, Syracuse, New York, USA

**Keywords:** halophiles, extremophiles, environmental microbiology

## Abstract

*Salimicrobium* sp. PL1-032A was isolated from Pearse Lakes, Western Australia. The sequenced genome consists of a single chromosome (2,705,688  bp) with a GC content of 47.2%. The isolation of *Salimicrobium* sp. PL1-032A contributes to the collection of culturable extremophiles and offers potential insight into the Pearse Lakes biome.

## ANNOUNCEMENT

*Salimicrobium* are typically Gram-positive, non-motile cocci, endospore-forming, moderate halophiles found in hypersaline lakes (1, 2). Rottnest Island (~30.4 km offshore from Perth, Western Australia) hosts athalassic soda-hypersaline lakes, including Pearse Lakes (3). Here, we report the complete genome sequence of a *Salimicrobium* sp. PL1-032A, isolated from Pearse Lakes in May 2022, when the lakes had a pH of 8.0 ± 0.01 and salinity of 20.1% ± 1.44%.

**Fig 1:**
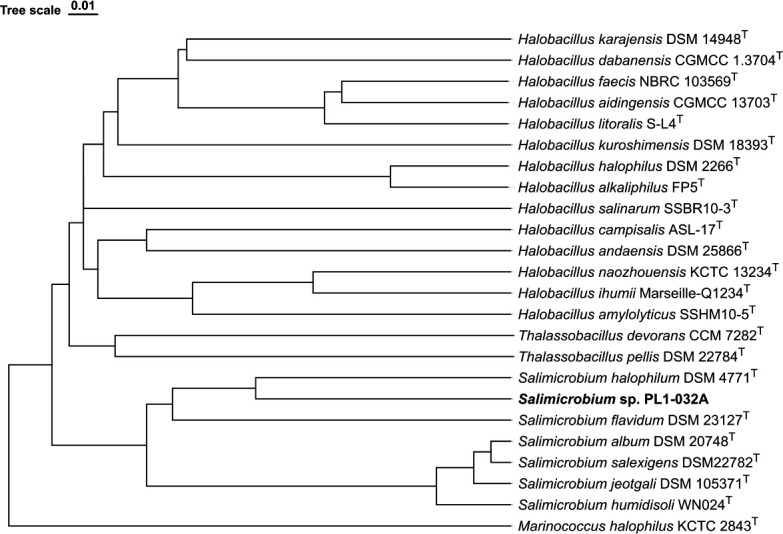
Dendro-unweighted pair group method with arithmetic mean (UPGMA) tree displaying the relatedness of *Salimicrobium* sp. PL1-032A to related species based on ANIb values. The ANIb values were generated using JSpeciesWS (11) and imported into DendroUPGMA, and the tree was constructed using a similarity matrix (Pearson’s correlation coefficient) within the algorithm (14). The tree was exported from MEGA11 in Newick format and imported into tvBOT (https://www.tvBOT.html) (15). The superscript T indicates type strains and *Marinococcus halophilus* KCTC 2843^T^ used as an outgroup (16).

Water samples were collected (S 32° 0′ 22.281″ E 115° 30′ 44.484″) and stored at 4°C before cultivation. Water samples (1,500 mL) were centrifuged at 4,500 × *g* for 10 minutes and streaked from the cell pellet on adapted lysogeny broth containing (per liter) 10.0 g bacto-tryptone, 5.0 g bacto-yeast extract, 150.0 g NaCl, and 2.4 g HEPES (pH 7.8) (4). Single colonies were re-streaked until pure cultures were obtained and cryopreserved (15% glycerol, −80°C). Genomic DNA (gDNA) was extracted from stationary-phase culture using the CTAB (2%) (5) and was sequenced using Oxford Nanopore Technology (ONT). The ONT library was prepared according to the ONT ligation native barcoding gDNA library protocol (SQK-NBD114.24) (https://nanoporetech.com/protocols) with a FLO-PRO114M flow cell (R10.4.1) on the PromethION 2 platform. Guppy [v6.5.7 (6)] was used for base-calling sequenced data with a read-pass-filter quality score cutoff value of 9 and a minimum length of 1,500 bp. Nineteen thousand two hundred twenty-seven reads were generated (146,068,268 bp) providing an average coverage of 54× and a *N*_50_ value of 14,731 bp determined with NanoStat [v1.6.0 (7)]. ONT long reads were assembled using Flye [v2.9.2 (8)] using default parameters, with nine iterations to provide a single circular chromosome (2,705,688 bp with 47.2% GC content). A quantitative genome assembly assessment was performed using BUSCO [v5.4.6 (9)] and CheckM [v1.2.2 (10)], providing a completeness score of 91.5% and 99.78% (0.22% contamination), respectively. Average nucleotide identity analysis using BLAST (ANIb) showed below the cutoff value (>95%) with the closest relative (83.4%) *Salimicrobium halophilum* DSM 4771^T^ (GCF_900100295.1) (11–13) (Fig. 1).

Gene calling and annotation of the generated chromosomal sequence were performed using the NCBI Prokaryotic Genome Annotation Pipeline (Table 1) [v6.6 (17)]. The isolation of *Salimicrobium* sp. PL1-032A and completion of the genome could provide knowledge about the extreme Pearse Lakes biome (18).

**TABLE 1:** General feature of the genome of *Salimicrobium* sp. PL1-032A (Accession: CP139874) from Prokaryotic Genome Annotation Pipeline

	Data from
Feature	GenBank annotation
Total no. of genes	2,795
No. protein-coding sequences	2,506
No. of rRNA operons	7
5s	7
16s	7
23s	7
No. of tRNA genes	67
No. of other RNA genes	4
Locus tag prefix	U0355_

## Data Availability

The *Salimicrobium* sp. PL1-022A whole-genome sequence (CP139874) has been deposited in the GenBank database (NCBI) under BioSample: SAMN38237613 within BioProject: PRJNA1039979. ONT long reads were deposited in SRA (SRR26991452).
